# 38. Sarcoidosis and pyoderma gangeronosum: two rare diseases occurring together

**DOI:** 10.1093/rap/rky034.001

**Published:** 2018-09-20

**Authors:** Naval Mendiratta, Shruti Bajad, Muzaffar Bindroo

**Affiliations:** 1 Rheumatology, Fortis Memorial REsearch Institute, Haryana, INDIA; 2 Rheumatology, Medanta the Medcity Hospital, Haryana, INDIA

**Introduction:** We present a case of unusual combination of sarcoidosis with refractory pyoderma gangrenosum. Pyoderma gangrenosum has been a reported with other autoimmune disorders like rheumatoid arthritis and ulcerative colitis, but it has hardly ever been reported with another rare disease like sarcoidosis. Here we present a case of 39 year old female, who was diagnosed and treated for sarcoidosis but later presented to our OPD with worsening skin lesions which were biopsied and diagnosed as pyoderma gangrenosum. Not only were they unusual, but they were even refractory to standard medical therapy.

**Case description:** A 39 year old female, was referred to our OPD for further management once the diagnosis of sarcoidosis was confirmed. Symptoms started in December 2014, when she had complaints of fever along with cough. Further evaluation was done and a chest CT revealed multiple mediastinal lymph nodes. Patient was started on empirical anti tubercular drugs (four drug regimen) ,which was given for six months . She felt better during the treatment course but after a period of two months, she started having again low grade fever with myalgias and arthalgias. Patient was admitted under internal medicine and was extensively evaluated. Repeat chest CT was suggestive of multiple mediastinal lymph nodes, with no shrinkage in size as compared to the initial one. Her erythrocyte sedimentation rate (ESR) and C-reactive protein (CRP) were still elevated (76 and 54) while the angiotensin converting enzyme (ACE) levels were slightly high(86). Endoscopic ultrasound-guided fine needle aspiration cytology (FNAC) was done which was suggestive of granulomatous inflammation (no evidence of necrosis, AFB stain negative). Considering the diagnosis of sarcoidosis, she was started on intramuscular methylprednisolone acetate 80 mg weekly for four weeks along with hydroxychloroquine. She reported improvement in fever and myalgias in the next visit and the inflammatory markers had normalised as well. But a month later, she presented to our OPD with new skin lesions over the arms and legs, one of which had appeared after minor trauma to the skin. Patient was admitted, and a deep skin biopsy was done from the ulcer site which was suggestive of pyoderma gangrenosum. There were no associated gastrointestinal symptoms or arthritis (the most common causes of pyoderma gangrenosum). The inflammatory markers at this stage were still normal (ESR 24 and CRP 5.0). She was started on oral corticosteroids (1 mg/kg) along with azathioprine (as steroid sparing drug). The patient had partial improvement but while tapering steroids to 20 mg/day, the lesions worsened and the new lesions appeared over the breasts, biceps area and the thighs. She was already experiencing steroid side effects related to a high dose of long duration. Biopsy was rechecked to confirm the diagnosis, but the conclusion remained the same. A therapeutic trial more of several additional weeks was planned before switching the medications. On the fourth follow-up visit, the skin lesions had worsened and began to increase in size over the thighs, breasts and the legs. She received a prolonged course of antibiotics along with daily dressings but all to no avail. Considering refractory disease, azathioprine was discontinued and patient was switched to injectable cyclophosphamide, according to literature for refractory pyoderma gangrenosum. She was given six monthly cycles, during which the patient showed significant improvement. But since the lesions persisted, we added colchicine 0.5 mg twice daily and gave two more doses of injectable cyclophosphamide (total 8 doses). The lesions began to heal after second dose but she needed prolonged injectable course to help the disease go in remission. The steroid could be tapered down to 5 mg/day. Currently, our patient is doing well with no new lesions. She has been kept on azathioprine maintenance therapy along with prednisolone 5 mg/day.

**Discussion:** Pyoderma gangrenosum is a rare disorder causing multiple non-healing ulcers over the limbs, aggravated by trauma. It is a neutrophilic dermatosis which has been associated mostly with inflammatory bowel disease and haematological malignancies, although most cases remain idiopathic. The association with sarcoidosis is a rare occurence. We did a review of the medical literature and found only four previously reported cases . The pathogenesis of pyoderma gangrenosum is immune mediated with predominant neutrophil infiltration. There has been an association with TNF-alpha, IL-8 and I-23 especially the variant associated with immunological diseases. Sarcoidosis is also an immune mediated disease with increased accumulation of CD 4 + T cells at the site of inflammation. The cytokine response in granulomatous inflammation is mediated by IL-2, TNF-alpha and interferon gamma. Skin manifestations are a known complication in sarcoidosis with manifestations like lupus pernio, erythrema nodousum, maculopapular rashes and keloid scars due to underlying inflammation. Pyoderma gangrenosum has not been reported due to the rarity of the cases being diagnosed. Both conditions have a common denominator of increasing skin lesions after injury, hence there can be a relation between the two. Our patient had a refractory pyoderma gangrenosum, which kept recurring upon tapering steroids. Her lesions had a predilection for areas of trauma, as the ulcers would appear on the sites she got injury with delayed healing. We did a literature search where cyclophosphamide and TNF Blockers has been recommended for refractory pyoderma gangrenosum. She showed a dramatic response to cyclophosphamide as after the first dose the wounds started to heal. After three doses of cyclophosphamide, she showed a much better response with larger lesions healing up as well. This case does give us a relationship between sarcoidosis and pyoderma gangrenosum. Unless one has a high index of suspicion, these cases may be overlooked. A chest roentgenogram should be recommended in all patients with idiopathic pyoderma gangrenosum.

**Key Learning Points:** This case does give us a relationship between sarcoidosis and pyoderma gangrenosum. Unless one has a high index of suspicion, these cases may be overlooked. A chest roentgenogram should be recommended in all patients with idiopathic pyoderma gangrenosum.

**Disclosure: N. Mendiratta:** None. **S. Bajad:** None. **M. Bindroo:** None.

